# Biomarkers in Rare Diseases

**DOI:** 10.3390/ijms22020673

**Published:** 2021-01-12

**Authors:** Bridget E. Bax

**Affiliations:** Institute of Molecular and Clinical Sciences, St. George’s University of London, London SW17 0RE, UK; bebax@sgul.ac.uk; Tel.: +44-2082666836

There is no single global definition of a rare disease, and for different geographical areas the definition is based on the disease occurrence in that population. In the USA, a rare disease is defined as a disorder that affects less than 200,000 individuals in the population, and in Europe it is a disease that affects less than five individuals in a population of 10,000. Despite the rarity of the individual diseases that fall under the geographical definitions, collectively these diseases are estimated to effect around 400 million people globally. Currently, 6000 to 8000 rare diseases have been identified, and new conditions are regularly being described in the literature. Despite incentives offered through orphan drug designation, as yet, only around 5% of these rare diseases have licenced treatments. Rare disease drug development is confounded by multiple challenges, and clinical trial failure is not uncommon. Regulatory approval of all drugs is based on a benefit–risk ratio, where benefit is measured using clinical trial endpoints which assess the way a patient feels, functions, or survives, and risk refers to the safety profile of the drug. Selecting appropriate clinical trial endpoints for rare diseases can be extremely challenging, and contributing to this are small patient populations, phenotypic heterogeneity, variable time frames for disease progression, incomplete knowledge of the disease pathophysiology or natural history, an absence of prior clinical studies, and non-existent validated disease-appropriate endpoints. Regulatory bodies recognise the need to accelerate rare disease drug development and may endorse the use of biomarkers or surrogate endpoints if validated endpoint measures are not available for the intended patient population. The definition of a biomarker is a biological characteristic that can be objectively measured and evaluated as a signal of normal biological or pathological processes and thus has the potential to improve diagnosis, predict disease manifestation, and monitor responses to therapeutic intervention. Technical advances in platforms employed for molecular analysis have permitted the identification of a wide range of candidate biomarkers for rare diseases, however, very few of these candidates are validated sufficiently for integration into the clinical management of patients.

The focus of this Special Issue is to provide a broad platform for original research and state-of-the-art reviews on novel or established proteomic, metabolomic, or transcriptomic biomarkers that contribute to the understanding of the underlying molecular mechanisms of rare diseases and/or that can be used for the diagnosis and prognosis of disease and individuals’ responses to therapies. The collection includes eight original research papers, one commentary, and six review articles from groups investigating biomarkers for a diverse range of rare diseases. 

Several articles focus on lysosomal storage disorders, with the interesting metabolomic study of Menkovic and co-workers recommending a biomarker panel rather than a single biomarker to overcome some of the diagnostic challenges and heterogeneous presentation of Gaucher disease [1]. In the second article from this group, Boutin and colleagues demonstrate a diurnal variation of disease biomarkers in the urine of patients with Fabry disease and recommend a morning collection of urine specimens for longitudinal evaluations [2]. The comprehensive review of Simonetta and colleagues discusses the potential of metabolic, proteomic, and transcriptomic profiles and their association with different organ systems and disease severity in Fabry disease. The authors cite small sample sizes as being a limiting factor in these studies and the need to validate correlations between scores of disease severity and biomarker levels in larger patient cohorts [3]. In their review, Showalter and co-workers explore the emerging and diverse roles of the phospholipid class bis(monoacylglycero)phosphate (BMP), which are localized almost exclusively within the membranes of late endosomal and lysosomal vesicles, and have been shown to be dysregulated not only in lysosomal storage disorders, but phospholipidosis, metabolic diseases, liver and kidney diseases, and neurodegenerative disorders. The authors recognise that an improved understanding of the enzymes involved in the catabolism and anabolism of BMP is required to clarify the functional significance of this class of phospholipid in the normal biological and pathological states [4].

Two contributions from Borrego’s group focus on Hirschsprung disease, a disorder caused by a defective proliferation, differentiation, survival, and/or migration of enteric precursor cells originating from the neural crest [5,6]. Current tests employed for the diagnosis of Hirschsprung disease are burdensome, and thus the availability of a simple disease biomarker would provide immense benefit to the patient and clinician. Using a combined experimental and computational approach, Villalba-Benito et al. report the dysregulation of five candidate genes related to *PAX*, which are potentially involved in the development of the enteric nervous system and implicated as the cause of disease onset [5]. In the group’s commentary, Torroglosa et al. report the dysregulation of three long non-coding RNAs (lncRNAs) in the enteric precursor cells of patients with Hirschsprung disease, compared to healthy controls [6]. lncRNAs are transcripts with a length greater than 200 nucleotides and have regulatory roles in gene expression at the epigenetic, transcriptional, translational, post-transcriptional, and post-translational levels in many biological processes. The authors propose the three transcripts (*SOCS2-AS*, *MEG3*, and *NEAT1*) as possible regulatory elements implicated in the onset of Hirschsprung disease and suggest that they could potentially serve as biomarkers of this disease.

Another study investigating the utility of lncRNAs as biomarkers is that of Skiriute and co-workers, who demonstrated that lncRNA *CASC2* was proportionally downregulated in progressed gliomas, while *miR-21* expression was inversely associated with *CASC2* expression, malignancy grade, and patient survival [7]. The authors conclude that *CASC2* and *miR-21* play antagonistic roles and potentially interact in glioma progression.

Two contributions in this Special Issue focus on the application of specific immune responses as biomarkers of disease. Spehner and co-workers analysed the impact of docetaxel, cisplatin, and 5-fluorouracil chemotherapy regimen and the prognostic value of adaptive immune responses and immunosuppressive cells in advanced anal squamous cell carcinoma (SCCA) patients who were included in two prospective clinical trials [8]. Through the measurement of T-cell responses against human papillomavirus (HPV)16-E6/E7 and anti-telomerase (hTERT)-antigens, they were able to demonstrate that whereas regulatory T-cells and monocytic-myeloid-derived suppressive cells (M-MDSC) accumulated in the peripheral blood of these patients, only high levels of M-MDSC were negatively correlated with hTERT adaptive immune responses and predicted poor prognosis. From these findings, the authors concluded that hTERT is a relevant antigen in HPV-driven SCCA disease and that the levels of M-MDSC influence TH1-adaptive immune responses and patient survival. In researching biomarkers of sarcoidosis, an inflammatory disease of unknown origin, Zhou and Arce provide an important review of the key players and biomarkers of the adaptive immune system in the pathogenesis of this disorder [9]. The authors highlight that there is no specific biomarker for sarcoidosis and that the majority of biomarkers examined to date remain unvalidated in the clinical setting or are insufficiently specific or sensitive for use in isolation for the purpose of clinical-decision making. They propose that the accuracy of diagnosis could be improved through the examination of signalling abnormalities and imbalances in B and T lymphocyte populations, and conclude these could potentially be used to detect more active or severe forms of sarcoidosis, predict the success of certain therapies, and explain disease etiology/mechanism.

An interesting contribution of Benabdelkamel and colleagues is their serum-based proteomics profiling study in adult patients with cystic fibrosis [10]. This disorder is caused by mutations in the cystic fibrosis transmembrane conductance regulator chloride channel gene and is the most common lethal autosomal recessive disorder in the Caucasian population. An ingenuity pathway analysis of the 134 differentially regulated proteins identified in the serum of these patients found that these proteins were related to inflammation and tissue repair, such as anti-proteases and complement factors. Transport proteins of vitamin A and D and lipoproteins were downregulated, suggesting possible explanations for their deficiencies in this disease. The authors concluded that although these proteomic studies have significantly enhanced the understanding of the complex pathogenesis of cystic fibrosis, additional studies are necessary to identify novel biomarkers.

Another study investigating inflammatory changes as a relevant biomarker of disease is that of Corey-Bloom and co-workers [11]. This group examined inflammation markers in the saliva and plasma of patients with Huntington’s disease, an inherited, progressive neurodegenerative disorder. Interestingly, salivary levels of Interleukin-6 (IL-6) were found to be significantly associated with prominent disease symptoms in Huntington’s disease mutation carriers, and were correlated to cognitive measures in healthy participants. Thus, the inflammatory change detected in peripheral saliva may be biologically relevant and mirror the neurodegenerative process occurring in the central nervous system. The authors conclude that although IL-6 elevation is unlikely to be specific to Huntington’s disease compared to other neurodegenerative diseases, the availability of a dependable salivary biomarker would meet the urgent need for a less invasive process for identifying and monitoring Huntington’s disease progression.

The contribution of Boziki and collaborators provides a review of the advances made in relation to biomarkers of rare neuroinflammatory diseases of the central nervous system [12]. The authors highlight the need for more accurate phenotype characterization, consensus guidelines, evidence-based definition of clinical outcomes, and the necessity for collaborations to enable a full characterization of the clinical, radiological, biological, and pathological spectrum of these syndromes.

Cerasuolo and co-workers used a combined molecular and bioinformatics approach to investigate the molecular basis of the genotype–phenotype correlation of Peutz–Jeghers syndrome, a disorder characterized by the development of noncancerous hamartomatous polyps in the gastrointestinal tract [13]. In the majority of cases, the disorder is caused by mutations in the tumour suppressor gene *STK11*. Early clinical diagnosis is often difficult due to its variable penetrance, and thus *STK11* genetic screening is recommended in suspected cases. The authors propose that splicing alterations contribute to the phenotypic variability and disease onset and highlight the importance of RNA analysis in the genetic testing of young patients.

Pasta and colleagues present a comprehensive review of 73 publications with the aim of categorizing molecular biomarkers for monitoring the progression of haemophilic arthropathy in patients with haemophilia A and B [14]. These X-linked bleeding disorders are characterized by recurrent hemarthroses leading to changes in the synovium and cartilage, and ultimately joint destruction. The availability of biomarkers that could signify disease at preclinical or asymptomatic phases, when the disease process is potentially reversible, would dramatically change the quality of life of these patients. From their review of the current literature, the authors conclude that although there is much interest in the search for biomarkers of hemarthrosis, their clinical use has little bearing on clinical practice because of the absence of standardization, and recommend that correlations are made with clinical and radiological parameters. 

The contribution of Doo and co-workers provides an interesting review of 28 articles relating to sudden sensorineural hearing loss, with the aim of evaluating the disease course and identifying a prognostic biomarker that could predict treatment outcome [15]. Although the authors identified that a good prognosis was associated with a range of measures, including low ratios of neutrophil/lymphocyte and monocyte/high-density lipoproteins, low monocyte counts, and low concentrations of fibrinogen, glycoprotein IIIa, and Tumor necrosis factor α, they were unable to draw any firm conclusions.

To conclude, this Special Issue highlights the diverse range of biomarkers that are under investigation for the screening of rare diseases and providing indicators of disease prediction, prognosis, and responses to treatment. One of the recurring recommendations is the need for biomarker standardization and the correlation of biomarkers with clinical and other measurable parameters. Further investigation into the underlying molecular mechanisms of disease pathology and the integration of biomarker studies into controlled clinical studies will permit significant advances in a field where there are currently many unmet needs. 
